# Targeting and Reprograming Cancer-Associated Fibroblasts and the Tumor Microenvironment in Pancreatic Cancer

**DOI:** 10.3390/cancers13040697

**Published:** 2021-02-09

**Authors:** Yoshiaki Sunami, Viktoria Böker, Jörg Kleeff

**Affiliations:** Department of Visceral, Vascular and Endocrine Surgery, Martin-Luther-University Halle-Wittenberg, University Medical Center Halle, 06120 Halle, Germany; viktoria.boeker@uk-halle.de (V.B.); joerg.kleeff@uk-halle.de (J.K.)

**Keywords:** pancreatic cancer, tumor microenvironment, cancer immunotherapy, reprogramming cancer-associated fibroblasts, mesenchymal stem cells, tumor-promoting cancer-associated fibroblasts, tumor-restraining cancer-associated fibroblasts

## Abstract

**Simple Summary:**

The tumor microenvironment plays a major role in the progression and drug resistance of pancreatic cancer. Cancer-associated fibroblasts are the major stromal cells and source of extracellular matrix proteins forming the dense stromal tumor microenvironment. Targeting cancer-associated fibroblasts has been deemed a promising therapeutic strategy. However, depleting cancer-associated fibroblasts may also have tumor-promoting effects due to their functional heterogeneity. It is therefore important to target selectively the tumor-promoting subtype of cancer-associated fibroblasts. Furthermore, deactivating fibroblasts, or reprograming tumor-promoting cancer-associated fibroblasts to tumor-restraining cancer-associated fibroblasts are considered as therapy for pancreatic cancer.

**Abstract:**

Pancreatic cancer is the fourth leading cause of cancer deaths in the United States both in female and male, and is projected to become the second deadliest cancer by 2030. The overall five-year survival rate remains at around 10%. Pancreatic cancer exhibits a remarkable resistance to established therapeutic options such as chemotherapy and radiotherapy, due to dense stromal tumor microenvironment. Cancer-associated fibroblasts are the major stromal cell type and source of extracellular matrix proteins shaping a physical and metabolic barrier thereby reducing therapeutic efficacy. Targeting cancer-associated fibroblasts has been considered a promising therapeutic strategy. However, depleting cancer-associated fibroblasts may also have tumor-promoting effects due to their functional heterogeneity. Several subtypes of cancer-associated fibroblasts have been suggested to exhibit tumor-restraining function. This review article summarizes recent preclinical and clinical investigations addressing pancreatic cancer therapy through targeting specific subtypes of cancer-associated fibroblasts, deprogramming activated fibroblasts, administration of mesenchymal stem cells, as well as reprogramming tumor-promoting cancer-associated fibroblasts to tumor-restraining cancer-associated fibroblasts. Further, inter-cellular mediators between cancer-associated fibroblasts and the surrounding tissue microenvironment are discussed. It is important to increase our understanding of cancer-associated fibroblast heterogeneity and the tumor microenvironment for more specific and personalized therapies for pancreatic cancer patients in the future.

## 1. Introduction

Pancreatic ductal adenocarcinoma (PDAC) is a devastating disease with an unfavorable outcome. Currently, pancreatic cancer is the fourth leading cause of cancer deaths in the United States both in female and male [1], and is projected to become the second deadliest cancer by 2030 [2]. Although a number of studies have shown significant progress in survival of pancreatic cancer patients by combination chemotherapies [3,4,5,6,7], the overall five-year survival rate stands still at 10% [1]. Pancreatic cancer exhibits a significant resistance to established therapeutic options such as chemotherapy and radiotherapy, in part due to the dense stromal tumor microenvironment [8]. Cancer-associated fibroblasts (CAF) are the major stromal cell type and source of extracellular matrix proteins shaping a physical and metabolic barrier thereby reducing therapeutic efficacy [9,10]. Targeting CAFs and disrupting ECM have been considered as promising therapeutic strategies. The cellular component of the desmoplastic stroma in pancreatic cancer is composed primarily of myofibroblasts with alpha-smooth muscle actin (α-SMA) expression [11]. Several studies have shown that the number of α-SMA-positive CAFs correlates with shorter overall survival in esophageal and pancreatic cancer patients [12,13], suggesting that depletion of α-SMA-positive cells can be a promising therapeutic strategy in cancer patients. However, deletion of α-SMA-positive cells in a pancreatic cancer preclinical mouse model (*Ptf1-Cre*; *lox-stop-lox-Kras^G12D/+^*; *Tgfbr2^lox/lox^*) leads to invasive, undifferentiated tumors that are more hypoxic and reduces animal survival [11]. In another pancreatic cancer mouse model called KPC (*Pdx1-Cre*; *lox-stop-lox-Kras^G12D/+^*; *lox-stop-lox-Trp53^R172H/+^*) [14], deletion of α-SMA-positive cells diminishes animal survival [11]. Focal adhesion kinase (FAK) is a key intracellular effector of ECM signaling, which is activated upon ECM-induced integrin receptor activation [15], suggesting that targeting FAK can be a therapeutic option in cancer. Deletion of FAK in the fibroblast-specific protein 1 (FSP-1)-positive cells in mice (*Fsp1-Cre*; *Fak^lox/lox^*) however leads to increased breast and pancreatic cancer growth [16]. Furthermore, low FAK expression is associated with shorter overall survival of breast and pancreatic cancer patients [16]. These studies imply that removal of cells based solely on positivity of α-SMA or FAK is not effective in limiting tumor aggressiveness. It has been demonstrated that static normal fibroblasts suppress polyoma virus-transformed tumor cells [17], suggesting that an innate function of fibroblasts is to protect against tumorigenesis [18]. Several subtypes of cancer-associated fibroblasts exhibit a tumor-restraining function [10,19]. Therefore, identification and better characterization of tumor-promoting CAF subtypes is important. Targeting inter-cellular mediators between cancer-associated fibroblasts and the surrounding microenvironment, deprogramming activated fibroblasts, administration of mesenchymal stem cells, as well as reprogramming tumor-promoting cancer-associated fibroblasts to tumor-restraining cancer-associated fibroblasts, have been considered as potential cancer therapeutic strategies.

## 2. Targeting Cancer-Associated Fibroblast Subtypes

**Figure 1:**
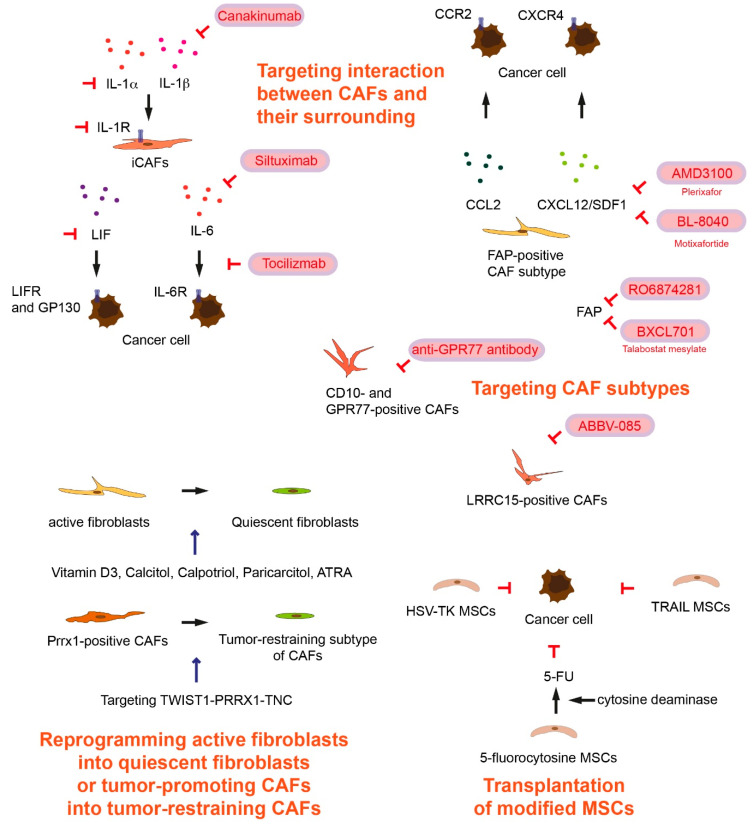
Therapeutic potential of targeting cancer-associated fibroblasts (CAFs), targeting interaction between CAFs and their surroundings, reprogramming active fibroblasts into quiescent fibroblasts or tumor promoting CAFs into tumor-restraining CAFs, and transplantation of modified mesenchymal stem cells (MSCs). The inhibition symbols are colored in red.

Fibroblast Activation Protein (FAP) is a type II transmembrane cell surface serine protease and shares high sequence homology with the Dipeptidyl Peptidase (DPP) 4 [20]. FAP is frequently (90%) expressed, predominantly in CAFs, in patients with pancreatic cancer [21]. High expression of FAP is associated with shorter overall survival and disease-free survival in pancreatic cancer patients. Global knockout of *Fap* delays pancreatic tumor progression and prolongs the survival in KPC mice [22]. In line with this, conditional ablation of FAP-positive cells (using Diphtheria Toxin Receptor (DTR) expressing selectively in FAP-positive cells) introduced into the KPC mouse line leads to inhibition of pancreatic tumor development [23]. A FAP monoclonal antibody conjugated to DM1, a cytotoxic drug tubulin-binding maytansinoid with antimitotic activity, called FAP5-DM1, inhibits tumor growth leading to complete regressions in lung, pancreas, head and neck in xenograft cancer models [24]. FAP-specific chimeric antigen receptor (CAR) T cells have been generated that present a specific targeting effect on FAP-positive cells [25,26]. Adoptive transfer of FAP-specific CAR T cells reduces FAP-positive stromal cells and concomitantly decreases tumor xenograft growth [25,26]. Adoptive transfer of anti-FAP CAR T cells inhibits the growth of pancreatic cancers in KPC mice [27]. However, in another study, infusion of FAP CAR T cells led to bone toxicity and cachexia in non-tumor bearing wild-type mice as well as in global *Rag1*-deficient mice bearing a stroma-rich pancreatic cancer xenograft with strong FAP-positivity [28]. Bone marrow stromal cells express FAP and therefore FAP-reactive T cells can recognize and kill the cells [28,29,30]. A phase 2 trial of FAP inhibition using a humanized monoclonal antibody sibrotuzumab failed due to ongoing tumor progression in colorectal cancer patients (NCT02198274) (Table 1) [31,32]. Targeting FAP-positive stromal cells by CAR T cells remains a promising therapeutic option. However, additional markers are further required to increase specificity for FAP-positive CAFs in pancreatic cancer. A phase 1/2 study with talabostat mesylate (BXCL701) (Figure 1) in combination with the anti-programmed cell death 1 (PD-1) monoclonal antibody pembrolizumab recruits prostate cancer patients to assess safety and efficacy of the combined treatment (NCT03910660) (Table 1). Talabostat is a specific inhibitor of dipeptidyl peptidases such as FAP [33]. Several clinical trials use RO6874281, FAP-targeted IL-2 variant, as a FAP inhibitor (Figure 1). RO6874281 carries an engineered IL-2 variant lacking binding ability to IL-2Rα. Affinity to IL-2Rβγ is retained, resulting in activation of CD8^+^ T cells and natural killer (NK) cells, but reduced activity on immunosuppressive regulatory T (Treg) cells. The antibody part of RO6874281 binds with high affinity to bind FAP, and mediate retention and accumulation in malignant lesions [34]. A phase 1 clinical study is currently ongoing for evaluating safety and therapeutic activity of RO6874281 in combination with pembrolizumab in patients with metastatic melanoma (NCT03875079) (Table 1). Another phase 1 study is ongoing to evaluate safety, pharmacokinetics, and therapeutic activity of RO6874281 as a single agent or in combination with trastuzumab or cetuximab for patients with breast cancer, head and neck cancer (NCT02627274) (Table 1). For patients with advanced/metastatic head and neck, esophageal, and cervical cancers, a phase 2 study evaluates the therapeutic activity of RO6874281 together with atezolizumab (also known as MPDL3280A, an engineered anti-PD-L1 antibody [35]), Gemcitabine and a mitotic spindle poison Vinorelbine (NCT03386721) (Table 1) [36]. For patients with metastatic pancreatic cancer, a phase 1/2 study of multiple immunotherapy-based treatment combinations is underway (NCT03193190) (Table 1).

FAP-positive CAFs attract Gr-1^+^/CD11b^+^ myeloid cells, and co-injection of FAP-positive CAFs and tumor cells into immunocompetent mice increases the frequency of infiltration of macrophages [37]. FAP-positive CAFs are a major source of chemokine CC-chemokine ligand 2 (CCL2) also known as monocyte chemoattractant protein-1 (MCP-1) (Figure 1). Administration of neutralizing anti-CCL2 antibody or CCL2 knockdown by shRNA blocks migration of Gr-1^+^/CD11b^+^ myeloid cells toward cell culture supernatants from FAP-positive CAFs [37]. The CCL2 and CC chemokine receptor type 2 (CCR2, MCP-1 receptor) axis is important for monocyte recruitment [38]. In global CCR2 knockout mice, FAP-positive CAFs fail to promote tumor growth in a liver tumor xenograft model [37]. In a doxycycline-inducible pancreatic cancer mouse model (*Ptf1-Cre*; *TetO-Kras^G12D/+^*; *Rosa26^rtTa-IRES-EGFP^*; *lox-stop-lox-Trp53^R172H/+^*), depletion of CD11b^+^ myeloid cells (*Itgam-DTR*) increased intratumoral CD8^+^ T cells [39,40]. It has been shown that CAFs expressing FAP suppress antitumor immunity [29]. FAP-positive CAFs express CXCL12 where T cell are excluded [23]. CXCL12 plays a role in tumoral immunosuppression. CXCL12, also known as stromal cell-derived factor 1 (SDF1) is the primary ligand for the C-X-C motif chemokine receptor 4 (CXCR4) (Figure 1). The CXCL12 and CXCR4 axis promotes pancreatic cancer development, invasion, and metastasis [41,42]. Targeting the CXCL12-CXCR4 axis by treatment with a specific CXCR4 inhibitor AMD3100 (Figure 1), also known as Plerixafor, reverses FAP-positive CAF-mediated immunosuppression, infiltration of CD8^+^ T cells into the tumor. Further AMD3100 treatment attenuates pancreatic cancer development in KPC mice [23]. The CXCL12-CXCR4 signaling pathway can recruit Treg cells [42]. However, Treg cells are not critically involved once immunosuppressive role of CXCL12 is neutralized. The precise roles of CXCL12 in immunosuppression still need to be clarified. A phase 1 study (CAM-PLEX study) for assessing the safety of continuous administration of Plerixafor (AMD3100) in patients with advanced pancreatic, ovarian and colon cancer has been completed (NCT 02179970) (Table 1). The results of the phase 1 study demonstrate that continuous administration of Plerixafor induces intratumoral CD8^+^ and natural killer (NK) cell accumulation in patients with pancreatic and colorectal cancer [43]. BL-8040 (motixafortide) is a CXCR4 antagonist, and a phase 2 study shows that BL-8040 increases CD8^+^ T cell tumor infiltration, decreases myeloid-derived suppressor cells and circulating regulatory T cells (COMBAT trial) (NCT02826486) (Table 1) [44]. These data suggest that alteration of tumor microenvironment by stromal and immune modulation is an appropriate therapeutic approach [32].

It has been shown that chemoresistant and chemosensitive tumors contain functionally distinct CAF subtypes. A study compared CAFs from chemoresistant breast cancer biopsies and CAFs from chemosensitive before neoadjuvant chemotherapy, and identified that cell-surface markers CD10 and G protein-coupled receptor 77 (GPR77) are upregulated in the CAFs from chemoresistant tumors [45]. CD10 is a zinc-dependent metalloproteinase, and GPR77, also known as C5L2, coded by the *C5AR2* gene, is an alternative receptor for complement C5a [46]. High expression of CD10- and GPR77-positive CAFs (Figure 1) are associated with docetaxel or cisplatin chemoresistance and shorter disease-free survival in breast and lung cancer patients [45]. CD10 and GPR77-positive CAFs sustain cancer stemness and promote tumor chemoresistance [45]. Treatment with a neutralizing anti-GPR77 antibody suppresses the activity of NF-κB, IL-6, and IL-8 secretion, abolishes establishment of breast cancer patient-derived xenograft in the immunocompromised mice, and restores docetaxel chemosensitivity [45].

Leucine-rich repeat containing 15 (LRRC15) is expressed on CAFs (Figure 1) in many solid cancers including pancreatic cancer as well as on a subset of cancer cells of mesenchymal origin [47,48]. A LRRC15-positive CAF subpopulation was identified in a pancreatic cancer mouse model (*Pdx1-Cre*; *lox-stop-lox-Kras^G12D/+^*; *p16/p19^lox/lox^*) [48]. Mouse tumor spheroids cultured with LRRC-positive CAFs grow larger than those in media alone, suggesting that LRRC-positive CAFs can directly enhance tumor growth [48]. ABBV-085 is a monomethyl auristatin E (MMAE)-containing antibody-drug conjugate directed against LRRC15, and ABBV-085 inhibits cancer cells tumor xenograft growth [47]. A phase 1 study with ABBV-085 to evaluate the safety and pharmacokinetics in patients with advanced solid tumors has been completed (NCT02565758) (Table 1). ABBV-085 was well-tolerated in the phase 1 study in patients with advanced sarcomas [49].

## 3. Targeting Interactions between Cancer-Associated Fibroblasts and Their Surrounding in Tumor Microenvironment

Another strategy is targeting signaling molecules involved in the interaction between CAFs and their surrounding environment. IL-6 from CAFs inhibits NK cell activity, and activates signal transducer and activator of transcription 3 (STAT3) in pancreatic cancer cells [50]. IL-6 and PD-L1 antibody (BioXCell) blockade combination therapy reduces tumor progression and extends overall survival in a murine pancreatic cancer model (*Pdx1-Cre*; *lox-stop-lox-Kras^G12D/+^*; *lox-stop-lox-Trp53^R270H/+^*; *Brca2^lox/lox^*) [51]. Siltuximab is an anti-IL-6 chimeric monoclonal antibody (Figure 1) [52]. A phase 1/2 study is currently recruiting metastatic pancreatic cancer patients for treatment with siltuximab and spartalizumab (a monoclonal antibody directed against human PD-1) (NCT04191421) (Table 2). Tocilizumab is a monoclonal antibody that competitively inhibits the binding of IL-6 to IL-6R (Figure 1) [53]. A phase 2 study (PACTO) investigates administration of tocilizumab in combination with gemcitabine and nab-paclitaxel for patients with unresectable pancreatic cancer (NCT02767557) (Table 2). Another phase 2 study with ipilimumab, nivolumab, tocilizumab and stereotactic body radiotherapy (SBRT) (TRIPPLE-R) has been launched for advanced pancreatic cancer patients (NCT04258150) (Table 2).

It has been demonstrated that IL-1 and transforming growth factor β (TGF-β) are key regulators of CAF heterogeneity [54]. The majority of fibroblasts in human pancreatic tumors as well as in tumors from KPC mice express FAP and low levels of α-SMA, whereas a subpopulation of FAP-positive cells exhibit elevated α-SMA expression called myofibroblastic CAFs (myCAFs) [55]. TGF-β downregulates IL-1R1 and promotes differentiation into myCAFs [54]. IL-1 activates NF-κB signaling and leukemia inhibitory factor (LIF) expression in a CAF subtype named inflammatory CAFs (iCAFs) [54]. Canakinumab is a human anti-IL-1β monoclonal antibody (Figure 1) [56]. A phase 1 study assesses safety and tolerability of canakinumab, spartalizumab, with the chemotherapy combination of gemcitabine and nab-paclitaxel in metastatic pancreatic cancer patients (NCT04581343) (Table 2). IL-1β is one of the IL-1 cytokines, canakinumab targets only one arm of the regulatory cytokines. Targeting IL-1α or IL-1R1 could also be therapeutic options. LIF further activates Janus kinase (JAK)/STAT signaling for IL-6 expression [54]. LIF can be a key paracrine factor produced by activated pancreatic stellate cells (PSCs) acting on cancer cells via activating STAT3 signaling [57]. In PSC-conditioned medium-stimulated human pancreatic cancer cells, LIF receptor (LIFR) and its co-receptor IL-6 signal transducer (IL6ST, also known as gp130) are only receptors identified as interacting partners with STAT3 (immunoprecipitation) [57]. Conditional deletion of LIFR leads to reduced pancreatic tumor progression and prolonged survival in a pancreatic cancer mouse model (*Pdx1-Cre*; *lox-stop-lox-Kras^G12D/+^*; *Trp53^lox/lox^*; *Lifr^lox/lox^*; *lox-stop-lox-Rosa26^Luc/Luc^*). Similar to genetic depletion of *Lifr*, administration of LIF neutralizing antibody increases overall survival and gemcitabine chemosensitivity in *Pdx1-Cre*; *lox-stop-lox-Kras^G12D/+^*; *Trp53^lox/lox^*; *lox-stop-lox-Rosa26^Luc/Luc^* mice [57].

## 4. Transplantation of Modified Mesenchymal Stem Cells

Mesenchymal stem cells (MSCs) exist in many tissues that have an important role in tissue regeneration [58]. However, MSCs (such as bone marrow-derived and adipose-derived MSCs) can migrate to tumors and differentiate into cancer-associated MSCs and tumor-promoting CAFs [10,58]. The complex cellular and molecular interaction, paracrine and reciprocal factors between MSCs and the surrounding microenvironment can lead to different function of MSCs and outcomes [10,58]. However, MSCs can be used for anti-cancer treatment because of their characteristic tendency to home to tumor sites. This property of MSCs makes these cells attractive candidates as drug delivery vehicles [58]. For treatment of gastrointestinal cancer, bone marrow-derived MSC-delivery of Herpes simplex virus type 1 thymidine kinase (HSV-TK) under the control of the CCL5-promotor has been used. After cell delivery, a cytotoxic drug ganciclovir is administered, which is phosphorylated and activated by HSV-TK for cancer cell death (Figure 1). This TREAT-ME1 study in phase 1/2 (NCT02008539) has been terminated and concluded as safe and tolerable (Table 3) [59,60,61]. Adipose-derived MSCs modified to express yeast cytosine deaminase co-administered with of the non-toxic prodrug 5-flurocytosine inhibit colon cancer cells tumor xenograft growth in immunocompromised mice [62]. Cytosine deaminase converts 5-flurocytosine to 5-fluorouracil (5-FU) (Figure 1) [62]. Co-administration of 5-flurocytosine and cytosine deaminase-expressing MSCs also inhibits melanoma cells tumor xenograft growth in nude mice [63].

MSCs can also be transfected with apoptosis-inducing factors (Figure 1). A phase 1/2 study with oncolytic measles virus encoding thyroidal sodium iodide symporter (MV-NIS)-infected MSCs recruits patients with recurrent ovarian, peritoneal, or fallopian tube cancer (NCT02068794) (Table 3). Oncolytic measles virus preferentially infects tumor cells and induces syncytia formation and apoptosis [64]. TNF-related apoptosis-inducing ligand (TRAIL)-transduced MSCs are resistant to TRAIL-induced apoptosis, due to low expression of the death receptors TRAIL-R1 and TRAIL-R2 in comparison with tumor cells [58]. A phase 1/2 study (NCT03298763, TACTICAL trial) is underway to recruit lung cancer patients using MSCs to deliver the TRAIL (Table 3). Taken together, several preclinical and clinical studies have demonstrated a great potential of MSCs in cancer therapy, yet it needs to be clarified whether MSCs keep their cellular status or transdifferentiate into tumor-promoting CAFs during therapies.

## 5. Reprogramming Active Fibroblasts into Quiescent Fibroblasts or Tumor-Promoting Cancer-Associated Fibroblasts into Cancer-Restraining Cancer-Associated Fibroblasts

Rather than complete depletion of CAF subtypes, reprogramming active fibroblasts into quiescent fibroblasts or the tumor-promoting subtype of CAFs into the tumor-restraining subtype of CAFs can be deemed to block pancreatic cancer progression and to render cancer cells more responsive to treatment (Figure 1) [10,19]. Vitamin D is a pluripotent fat-soluble prohormone and plays a critical role in calcium homeostasis, cell differentiation, proliferation as well as apoptosis. Furthermore, vitamin D deficiency is associated with increased risk of developing several types of cancer [65]. Epidemiological cohort studies have shown that higher intake of Vitamin D reduces pancreatic cancer risk [66]. Calcitriol (1α,25-dihydroxyvitamin D_3_, 1,25(OH)_2_D_3_) enhances gemcitabine efficiency to inhibit pancreatic cancer xenografts [67]. Vitamin D_3_ binds with Vitamin D receptor (VDR) and retinoid X receptor (RXR) to form a heterodimer complex regulating downstream signaling pathways [65]. Activation of VDR in PSCs by calcipotriol (vitamin D_3_ analog) attenuates inflammation and fibrosis. Furthermore, administration of calcipotriol in combination with gemcitabine enhances survival of KPC mice [68]. High VDR expression in CAFs is associated with longer overall survival and progression-free survival in colorectal cancer patients [69]. Currently several clinical studies with high-dose of vitamin D_3_ or paricalcitol (analog of the active form of vitamin D_2_) [70] are underway. A phase 3 study with high-dose vitamin D_3_ for treating pancreatic cancer patients has been launched (NCT03472833) (Table 4). Further an early phase 1 study with paricalcitol/gemcitabine/Nab-paclitaxel/Nivolumab for resectable pancreatic cancer patients (NCT03519308), and a phase 1 study with paricalcitol/5-FU/leucovorin/liposomal irinotecan for advanced pancreatic cancer (NCT03883919) have been initiated (Table 4). A phase 2 study with paricalcitol in combination with pembrolizumab has been completed (NCT03331562). Other phase 2 studies with paricalcitol/gemcitabine/Nab-paclitaxel (NCT04617067) or with paricalcitol/gemcitabine/hydroxychloroquine/Nab-paclitaxel (NCT04524702) are currently recruiting pancreatic cancer patients (Table 4).

Retinoids are natural and synthetic vitamin A derivatives such as all-trans retinoic acid (ATRA), 9-*cis* retinoic acid, and 13-*cis* retinoid acid, control cellular differentiation, growth, and apoptosis. ATRA activates retinoic acid receptors and is the principal activator of retinoic acid signaling [71]. ATRA induces quiescence of PSCs [72]. Quiescent PSCs produce Secreted Frizzled Related Protein 4 (SFRP4), which sequesters Wnt molecules and inhibits Wnt-meditated signal transduction. ATRA administration leads to a reduction of activated stroma as well as reduction of cancer cell proliferation in KPC mice. Further ATRA treatment increases apoptosis of cancer cells associated with a decrease in nuclear β-catenin and increase in stromal SFRP4 in KPC mice [72]. The combination of gemcitabine and ATRA results in inhibition of tumor growth in gemcitabine-resistant pancreatic cancer xenograft in mice. In pancreatic tumor xenograft with high fatty acid binding protein 5 (FABP5) expression and no cellular retinoic acid binding protein 2 (CRABP2) expression is resistant to the treatment with ATRA [73]. FABP5 delivers retinoic acid to peroxisome proliferator activated receptor β/δ (PPARβ/δ), while its homolog CRABP2 delivers retinoic acid to retinoic acid receptor (RAR). Both FABP5 and CRABP2 localize in the cytoplasm in the absence of ligand. Upon binding retinoic acid, they translocate into the nucleus to form a complex with either PPARβ/δ or RAR. PPARβ/δ or RAR forms heterodimer with RXR to regulate gene expression [74]. Heat shock protein 47 (HSP47) is a collagen-specific molecular chaperon that is required for the proper folding and secretion of collagen [75]. HSP47 enhances deposition of ECM proteins and promotes cancer progression in a breast cancer xenograft [76]. Co-delivery of ATRA and HSP47 siRNA, based on PEGylated polyethylenimine-coated gold nanoparticles system, induces PSC quiescence, thereby promoting drug delivery and enhancing the anti-tumor efficacy of gemcitabine in pancreatic cancer xenografts [75]. Juxtatumoral compartments of pancreatic cancer patient samples contain increased numbers of myeloperoxidase- and CD68-positive cells but less CD8-positve cells than in pan-stromal compartments. Pancreatic cancer patients with high density of CD8-positive T cells in the juxtatumoral compartment exhibit longer survival. ATRA administration increases CD8-positive T cells in juxtatumoral compartment in KPC mice [77]. A phase 1 clinical trial with ATRA in combination with gemcitabine and Nab-Paclitaxel (STARPAC study) has been completed (NCT03307148) (Table 4) [78]. A subsequent phase 2 study (ATRA/gemcitabine/Nab-paclitaxel) (STARPAC2) is currently underway (NCT04241276) (Table 4).

The homeodomain transcription factor paired-related homeobox 1 (PRRX1) regulates epithelial to mesenchymal transition (EMT) [79], and considered a driver of cellular plasticity during pancreatic ductal development, acinar-to-ductal metaplasia (ADM) formation and carcinogenesis [80]. One of two major isoforms of PRRX1, PRRX1b promotes EMT (de-differentiation), invasion, and tumor differentiation while PRRX1a stimulates mesenchymal to epithelial transition (MET) (re-differentiation), metastatic outgrowth as well as tumor differentiation [81]. These two isoforms of PRRX1 have therefore a reciprocal relationship in regulating cellular plasticity and regulate tumor progression at different stages [81]. In *Pdx1-Cre*; *lox-stop-lox-Kras^G12D/+^*; *Ink4a^lox/+^* murine pancreatic cancer and in pancreatic cancer patient specimens, PRRX1 is expressed in CAFs. High expression levels of PRRX1 in CAFs are associated with squamous subtypes of pancreatic cancer [82]. It has been shown that patients with the squamous subtype have shorter median survival [83]. PRRX1-deficient CAFs display attenuated cellular plasticity and differentiate into myCAFs [82]. Although it cannot be concluded that α-SMA-positive cells are simply tumor-restraining CAFs (see the Section 1), targeting PRRX1 can be a promising strategy for reprograming tumor-promoting subtype of CAFs to tumor-restraining subtype of CAFs. Twist family basic helix-loop-helix transcription factor (TWIST1) induces trans-differentiation of quiescent fibroblasts to CAFs [84]. TWIST1 is highly expressed in CAFs and a positive regulator of PRRX1, which subsequently increases the expression of Tenascin C (TNC). By forming a positive feedback loop, TNC increases TWIST1 expression [85]. Targeting TWIST1-PRRX1-TNC is hence a promising therapeutic target to deprogram activated fibroblasts or reprogram tumor-promoting CAFs to tumor-restraining CAFs.

## 6. Conclusions

Currently, various preclinical and clinical strategies have been developed to target subpopulations of tumor-promoting CAFs. Some of the most commonly used CAF markers are expressed on other cell types leading to side effects of CAF-targeted therapy. Further identification of tumor-promoting CAF subpopulations and specific markers are important to develop precise targeting strategies against the tumor-promoting CAF subtypes. Another strategy of pancreatic cancer therapy is targeting signaling molecules involved in the interaction between CAFs and their surrounding environment. Underlying mechanisms for CAF accumulation in cancers and identification of CAF subsets populating the tumor microenvironment would help in establishing therapeutic strategies to reprogram the tumor-promoting microenvironment into a tumor-suppressive microenvironment. Reprogramming active fibroblasts into quiescent fibroblasts or tumor-promoting subtype of CAFs into tumor-restraining subtype of CAFs can be used to block pancreatic cancer progression, to reprogram the tumor microenvironment, and to render cancer cells more responsive to treatments.

## Figures and Tables

**Table 1:** Overview of several clinical trials targeting fibroblast activation protein, CXCL12 and CXCR4 axis, and LRRC15.

Intervention/Treatment	Condition or Disease	NCT Number	Stage of Clinical Trial	Recruitment Status (Recruiting, Completed, not yet Recruiting. Last Update)	Last Update
BIBH1 (Sibrotuzumab)	Metastatic colorectal cancer	NCT02198274	Phase 2	Completed	23 July 2014
Talabostat mesylate (BXCL701) Pembrolizumab	Prostate cancer	NCT03910660	Phase 1/2	Recruiting	5 November 2020
RO6874281 Atezolizumab (MPDL3280A), an engineered anti-PD-L1 antibody Gemcitabine Vinorelbine	Advanced/Metastatic head and neck, esophageal and cervical cancers	NCT03386721	Phase 2	Recruiting	27 April 2020
RO6874281 with Trastuzumab or Cetuximab	Solid tumor Breast cancer Cancer of head and neck	NCT02627274	Phase 1	Recruiting	14 October 2020
RO6874281 Pembrolizumab	Metastatic melanoma	NCT03875079	Phase 1	Recruiting	9 October 2020
Nab-Paclitaxel Gemcitabine Oxaliplatin Leucovorin Fluorouracil Atezolizumab Cobimetinib PEGGH20 BL-8040 Selicrelumab Bevacizumab RO6874281 AB928 Tiragolumab Tocilizumab	Metastatic pancreatic adenocarcinoma	NCT03193190	Phase 1,2	Recruiting	27 October 2020
Cemiplimab (REGN-2810, Libtayo) Plerixafor (AMD3100, Mozobil)	Metastatic pancreatic cancer	NCT04177810	Phase 2	Recruiting	9 November 2020
Plerixafor (Mozobil)	Metastatic pancreatic cancer Metastatic colorectal cancer Ovarian Serous Adenocarcinoma	NCT02179970 (CAM-PLEX)	Phase 1	Completed	23 July 2019
BL-8040 Pembrolizumab Onivyde/5-FU/leucovorin	Metastatic pancreatic cancer	NCT02826486 (COMBAT)	Phase 2	Active, not recruiting	9 June 2020
ABBV-085	Advanced solid tumors	NCT02565758	Phase 1	Completed	5 April 2019

**Table 2:** Overview of clinical trials with targeting interaction between tumor-promoting cancer-associated fibroblasts and surrounding microenvironment.

Intervention/Treatment	Condition or Disease	NCT Number	Stage of Clinical Trial	Recruitment Status (Recruiting, Completed, not yet Recruiting. Last Update)	Last Update
Siltuximab Spartalizumab	Metastatic pancreatic cancer	NCT04191421	Phase 1,2	Recruiting	20 August 2020
Tocilizumab Gemcitabine Nab-Paclitaxel	Unresectable pancreatic cancer	NCT02767557 (PACTO)	Phase 2	Recruiting	21 April 2020
Ipilimumab Nivolumab Tocilizumab SBRT	Pancreatic cancer	NCT04258150 (TRIPPLE-R)	Phase 2	Recruiting	21 April 2020
Canakinumab Spartalizumab Nab-paclitaxel Gemcitabine	Metastatic pancreatic cancer	NCT04581343 (PanCAN-SR1)	Phase 1	Recruiting	20 October 2020

**Table 3:** Overview of clinical trials with transplantation of mesenchymal stem cells.

Intervention/Treatment	Condition or Disease	NCT Number	Stage of Clinical Trial	Recruitment Status (Recruiting, Completed, not yet Recruiting. Last Update)	Last Update
MSC_apceth_101	Advanced gastrointestinal cancer	NCT02008539	Phase 1, 2	Terminated	27 March 2017
Oncolytic measles virus encoding thyroidal sodium iodide symporter infected MSCs	Recurrent ovarian, peritoneal or fallopian tube cancer	NCT02068794	Phase 1, 2	Recruiting	3 December 2020
MSCTRAIL	Lung cancer	NCT03298763 (TACTICAL)	Phase 1, 2	Recruiting	31 March 2020

**Table 4:** Overview of clinical trials for reprogramming active fibroblasts into quiescent fibroblasts or tumor-promoting cancer-associated fibroblasts into tumor-restraining cancer-associated fibroblasts.

Intervention/Treatment	Condition or Disease	NCT Number	Stage of Clinical Trial	Recruitment Status (Recruiting, Completed, not yet Recruiting. Last Update)	Last Update
High-dose Vitamin D_3_	Pancreatic cancer	NCT03472833	Phase 3	Recruiting	14 September 2020
Pembrolizumab paricalcitol	Pancreatic cancer, advanced pancreatic cancer, metastatic pancreatic cancer	NCT03331562	Phase 2	Completed	8 October 2020
Nivolumab Nab-Paclitaxel Gemcitabine Paricalcitol	Resectable pancreatic cancer	NCT03519308	Early Phase 1	Recruiting	July 30, 2020
Paricalcitol Gemcitabine and Nab-paclitaxel	Advanced pancreatic cancer	NCT04617067	Phase 2	Recruiting	9 November 2020
5-FU Leucovorin Liposomal irinotecan Paricalcitol	Advanced pancreatic cancer	NCT03883919	Phase 1	Recruiting	27 August 2020
Gemcitabine Hydroxychloroquine Nab-paclitaxel Paricalcitol	Advanced or metastatic pancreatic cancer	NCT04524702	Phase 2	Recruiting	8 October 2020
ATRA Gemcitabine Nab-paclitaxel	Pancreatic cancer	NCT03307148 (STAR_PAC)	Phase 1	Completed	22 January 2020
ATRA Gemcitabine Nab-paclitaxel	Pancreatic cancer	NCT04241276 (STARPAC2)	Phase 2	Not yet recruiting	27 January 2020
